# Supplementary material for: Incidence and survival of pediatric and adult hepatocellular carcinoma, United States, 2001–2020
Source: medRxiv. 2024 Apr 5:2024.03.25.24304564. Preprint. [Version 2] doi: 10.1101/2024.03.25.24304564 (PMC11023662; doi:10.1101/2024.03.25.24304564)
Supplement: Supplement 2 [file NIHPP2024.03.25.24304564v2-supplement-2.pdf]

medRxiv preprint doi: https://doi.org/10.1101/2024.03.25.24304564; this version posted April 5, 2024. The copyright holder for this preprint (which was not certified by peer review) is the author/funder, who has granted medRxiv a license to display the preprint in perpetuity.
This article is a US Government work. It is not subject to copyright under 17 USC 105 and is also made available for use under a CC0 license.

eFigure 1

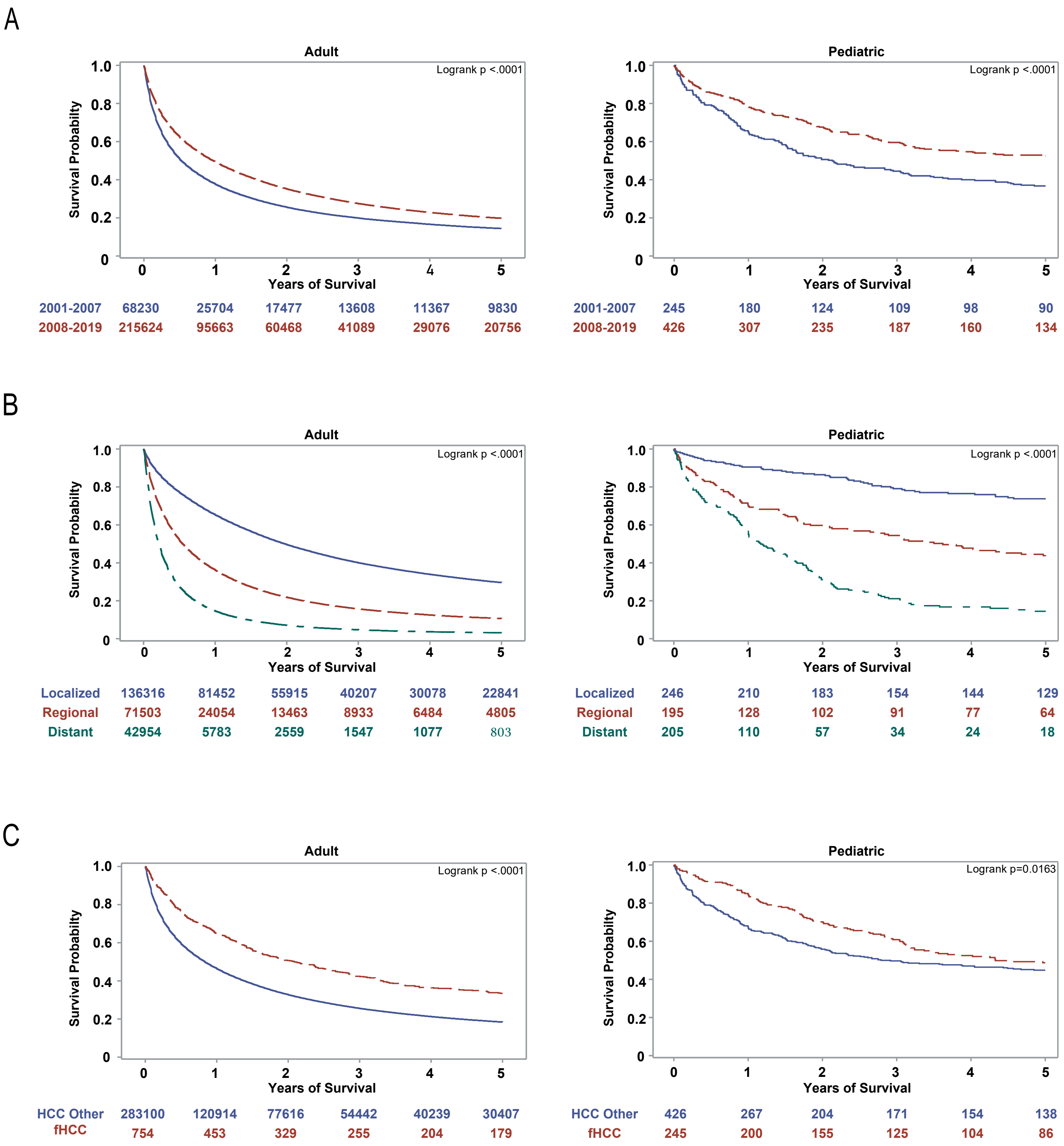

eFigure 1 5-year overall survival of adult (left) and pediatric (right) patients with hepatocellular carcinoma. A. Diagnosis year. B. Stage. C. Histolo
Abbreviation: fibrolamellar hepatocellular carcinoma (fHCC), hepatocarcinoma (HCC).

medRxiv preprint doi: https://doi.org/10.1101/2024.03.25.24304564; this version posted April 5, 2024. The copyright holder for this preprint (which was not certified by peer review) is the author/funder, who has granted medRxiv a license to display the preprint in perpetuity.
This article is a US Government work. It is not subject to copyright under 17 USC 105 and is also made available for use under a CC0 license.

eFigure 2

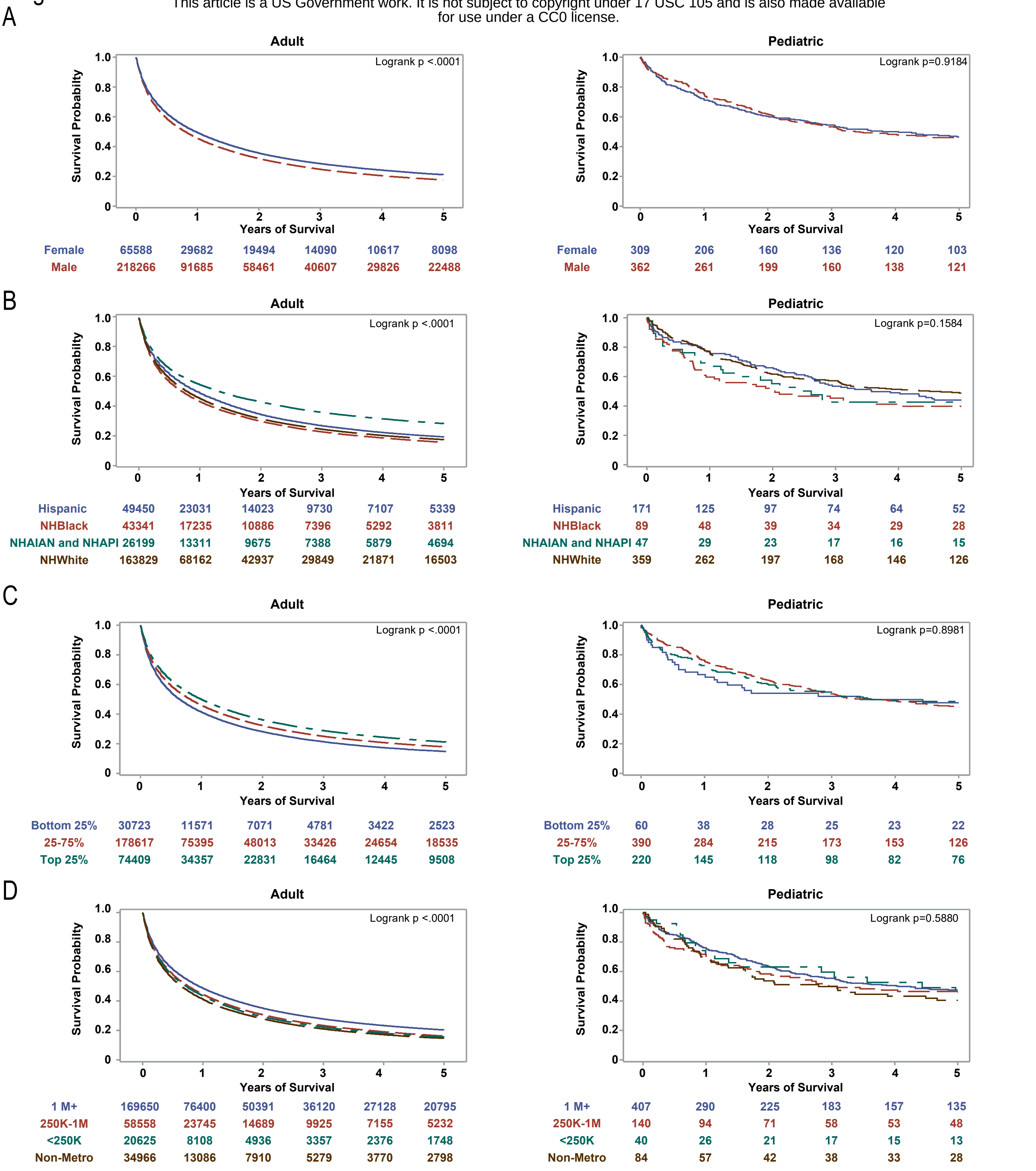

eFigure 2 5-year overall survival of adult (left) and pediatric (right) patients with hepatocellular carcinoma. A. Sex. B. Race and ethnicity. C. Socioeconomic status by county. D. Metropolitan status. Abbreviation: metropolitan (metro), Non-Hispanic (NH).
